# Prognostic Role of Combined EGFR and Tumor-Infiltrating Lymphocytes in Oral Squamous Cell Carcinoma

**DOI:** 10.3389/fonc.2022.885236

**Published:** 2022-07-25

**Authors:** Wattawan Wongpattaraworakul, Katherine N. Gibson-Corley, Allen Choi, Marisa R. Buchakjian, Emily A. Lanzel, Anand Rajan KD, Andrean L. Simons

**Affiliations:** ^1^ Department of Oral Pathology, Radiology, and Medicine, College of Dentistry, University of Iowa, Iowa City, IA, United States; ^2^ Department of Pathology, College of Medicine, University of Iowa Hospitals and Clinics, Iowa City, IA, United States; ^3^ Department of Pathology, Microbiology and Immunology, Vanderbilt University Medical Center, Nashville, TN, United States; ^4^ Department of Otolaryngology – Head and Neck Surgery, University of Iowa Hospitals and Clinics, Iowa City, IA, United States; ^5^ Holden Comprehensive Cancer Center, University of Iowa Hospitals and Clinics, Iowa City, IA, United States; ^6^ Iowa City Veterans Affairs Health Care System, Iowa City, IA, United States

**Keywords:** OSCC, EGFR, CD3, TIL, microarray, biomarker

## Abstract

**Background:**

Epidermal growth factor receptor (EGFR) is well known as a general prognostic biomarker for head and neck tumors, however the specific prognostic value of EGFR in oral squamous cell carcinoma (OSCC) is controversial. Recently, the presence of tumor-infiltrating T cells has been associated with significant survival advantages in a variety of disease sites. The present study will determine if the inclusion of T cell specific markers (CD3, CD4 and CD8) would enhance the prognostic value of EGFR in OSCCs.

**Methods:**

Tissue microarrays containing 146 OSCC cases were analyzed for EGFR, CD3, CD4 and CD8 expression using immunohistochemical staining. EGFR and T cell expression scores were correlated with clinicopathological parameters and survival outcomes.

**Results:**

Results showed that EGFR expression had no impact on overall survival (OS), but EGFR-positive (EGFR+) OSCC patients demonstrated significantly worse progression free survival (PFS) compared to EGFR-negative (EGFR-) patients. Patients with CD3, CD4 and CD8-positive tumors had significantly better OS compared to CD3, CD4 and CD8-negative patients respectively, but no impact on PFS. Combined EGFR+/CD3+ expression was associated with cases with no nodal involvement and significantly more favorable OS compared to EGFR+/CD3- expression. CD3 expression had no impact on OS or PFS in EGFR- patients. Combinations of EGFR/CD8 and EGFR/CD4 expression showed no significant differences in OS or PFS among the expression groups.

**Conclusion:**

Altogether these results suggest that the expression of CD3+ tumor-infiltrating T cells can enhance the prognostic value of EGFR expression and warrants further investigation as prognostic biomarkers for OSCC.

## Introduction

Oral cancer is the most common malignancy of the head and neck region (1, 2). More than 90% of all oral cancers are oral squamous cell carcinomas (OSCCs) (2) and arise in the tongue, floor of the mouth, palate, and labial and buccal mucosa (3). Despite the anatomical accessibility of OSCCs and advances in cancer diagnosis and treatment, the 5-year overall survival rate has remained at less than 50% for the last three decades (4). The main treatment approach for OSCC is surgery with post-operative radiotherapy (RT) with or without adjuvant systemic therapy (chemotherapy and/or targeted therapy). However, OSCC patients have a high risk of tumor recurrence and the main disease-related mortality in OSCC patients is due to locoregional failure (5). At this time, there are no prognostic or predictive molecular biomarkers used clinically for OSCCs. Treatment decisions depend primarily on tumor site, TNM classification, and clinicopathological parameters (6) which do not consistently predict patient prognosis (7, 8). There is an urgent need for more biomarker studies that will aid in rapid risk assessment and guide treatment decisions for OSCC patients

Epidermal growth factor receptor (EGFR) is a receptor tyrosine kinase in the ErbB family of receptors (9, 10). EGFR is involved in multiple complex pathways in cell regulation including embryogenesis, tissue regeneration and homeostasis (11–13). Dysregulation of EGFR expression/signaling in oral epithelial cells can lead to the development and progression of OSCCs (14–18). As a result, EGFR when measured by quantitative and semiquantitative immunohistochemistry (IHC) (19–22), is generally known as an independent prognostic factor for tumor recurrence in OSCC patients. However, the prognostic accuracy of EGFR expression is unreliable and somewhat controversial due to the lack of EGFR expression in many tumors in the head and neck region (23) and a number of conflicting reports demonstrating no prognostic value (24–31). Because of this, EGFR expression is not routinely tested for in the clinical setting for OSCC (28, 31–36) despite its established role in tumor aggressiveness.

Recently, growing evidence for the interaction of tumor and immune cells in tumor growth, recurrence and progression has emerged (37–41). OSCCs due to its unique anatomical location are characterized by an abundant infiltrate of immune cells (42). However, depending on the make-up and location of the immune cells in the tumor microenvironment (TEM), tumor-supporting outcomes are possible that contribute to immune escape (43, 44). Cells that contribute to immune escape mechanisms include tumor-associated macrophages (45), regulatory dendritic cells (DCs) (46), T regulatory cells (Tregs) (47) and myeloid-derived suppressor cells (MDSCs) (48, 49). On the other hand, in many cancers including OSCCs, an active anti-tumor immune response is often reflected by the abundance of tumor-infiltrating lymphocytes (TILs) such as CD8+ T cells (41, 50, 51) and is correlated with favorable prognosis (37–40). Tumor-infiltrating CD4+ T cells have recently been correlated with favorable prognosis in OSCCs (52) however CD4+ T cells consist of several subpopulations (including Tregs) and CD4 expression as a prognostic biomarker in OSCCs and HNSCCs is controversial (40, 53–55). Together the assessment of TILs is promising as a prognostic tool for OSCCs.

Given that the presence of TILs provides important information regarding overall prognosis in OSCCS, and EGFR expression provides valuable information regarding the risk of tumor recurrence/progression, the goal of this study is to investigate if TIL expression would enhance the prognostic value of EGFR in OSCC patients.

## Materials and Methods

### Tissue Microarrays

Formalin-fixed paraffin-embedded tumor samples of patients were obtained from 266 surgical resection specimens of OSCC spanning 10 years of time (2005-2014) from the archives of the Department of Pathology at the University of Iowa Hospitals and Clinics. Cases were chosen selectively to ensure patients with no history of radiation or chemotherapy and to ensure a mixture of patients regarding recurrent status, node metastases, margin status, age, and smoking status. After excluding cases with unavailable tissue blocks, 146 cases were included in the current study. TMAs were constructed using 3-6 morphologically representative areas (tumor and stroma). Sections (4μm) were obtained from the TMAs on poly-L-lysine-coated glass slides. Routine hematoxylin-and eosin (H&E) sections were reviewed to confirm the original diagnosis. Subject clinicopathological characteristics considered were age, sex, smoking history, tumor site, T stage, N stage, differentiation, and presence of perineural invasion, lymphovascular invasion, bone invasion and local recurrence. Clinicopathological characteristics were obtained from medical records where tumor microscopic features and TNM stage had been previously evaluated by board certified pathologists. The TNM status was based on the American Joint Committee on Cancer 7 (AJCC7).

### Immunohistochemistry

Antigen retrieval was performed on freshly cut sections in a decloaking chamber for 5 min at 125°C in TRIS buffer (pH 9.0). Endogenous peroxidase was blocked by incubation with 3% peroxide at room temperature for 8 min. IHC was performed with the following antibodies: EGFR (H11, Dako) at 1:200 dilution, CD3 (Dako A0452) at 1:200 dilution, CD4 (Novocastra NCL-L-CD4-368) at 1:100 dilution, and CD8 (Dako M7103) at 1:100 dilution. Bound antibody was detected using the HRP-DAB Cell & Tissue Staining Kit. All slides were counterstained with hematoxylin.

### Quantification of EGFR and TIL Staining:

EGFR immunostaining was evaluated by semiquantitative scoring (score 0-3) where 0 represents staining in <10% neoplastic cells and 1, 2, and 3 representing weak, moderate, and strong staining in >10% neoplastic cells according to Gamboa-Domingez and colleagues (56). Immunostaining scores of 3 and 2 were designated as EGFR-positive (EGFR+) and scores of 1 and 0 were designated as EGFR-negative (EGFR-). CD3+, CD8+, CD4+ and FoxP3+ TILs were evaluated based on the density of positive inflammatory cells (score 0-3): 0 represents none or very few positive cells; 1 represents single isolated positive cell or small aggregates of positive cells 2-4 cells; 2 represents discrete nodules or aggregates of positive cells more than 2-4 cells; 3 represents bands or continuous aggregates of positive cells. For CD3, the immunostaining scores of 3 and 2 were designated as high CD3 (CD3+), while scores of 1 and 0 were designated as low CD3+ (CD3-). Due to low number of specimens that received scores of 3 (n=2) and 2 (n=11) for CD8, the immunostaining scores of 3, 2 and 1 were designated as CD8+, while a score of 0 were designated as CD8-. For CD4, immunostaining scores of 3 and 2 were designated as high CD4 (CD4+), while scores of 1 and 0 were designated as low CD4 (CD4-). Immunoexpression of EGFR, CD3, CD4 and CD8 was scored by 2 pathologists. In the event of disagreement, a consensus score was given. Each tissue core received an individual score, and the average score was calculated from all the tissue cores that were from the same case and used as a final score. Very few HPV+ OSCC cases were present in the patient cohort and were excluded from the study.

### Statistical Analysis

Power for a sample size of 146 cases was estimated at 95.2% calculating from previous work (57). The association between expression scores and patient clinicopathological characteristics were analyzed by Chi-square test. Survival outcomes differences were plotted using the Kaplan-Meier method while estimates for the group hazard ratios were obtained using Cox proportional hazards (PH) modeling. Overall survival (OS) is defined as the length of time (in months) from the date of diagnosis that the patients remain alive. Progression free survival (PFS) is defined as the time from diagnosis to disease progression or death (in months) from any cause. All testing was performed on the univariate level and unadjusted for multiple comparisons. Differences between survival curves were compared using the log-rank test. A p-value below 0.05 was considered statistically significant. GraphPad Prism 8.1.2 was utilized for data analysis.

## Results

### Clinicopathological Characteristics

**Figure 1:**
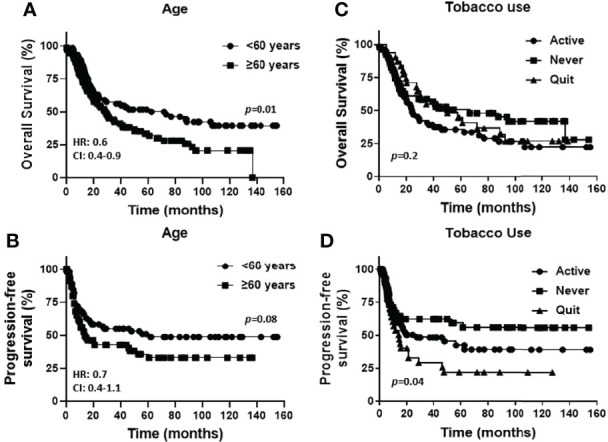
Prognostic impact of age and smoking history in OSCCs. Shown are Kaplan-Meier estimates of the overall survival **(A, C)** and progression free survival **(B, D)** of OSCC patients stratified by age [< 60 years or ≥ 60 years, **(A, B)**] and tobacco use history [active, never or quit, **(C, D)**]. HR: hazard ratio, CI: 95% confidence interval.

**Figure 2:**
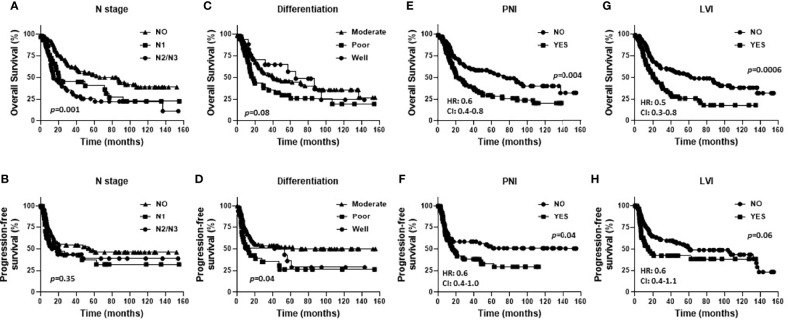
Prognostic impact of pathological factors in OSCCs. Shown are Kaplan-Meier estimates of overall survival **(A, C, E, G)** and progression free survival **(B, D, F, H)** of OSCC patients stratified by N stage **(A, B)**, differentiation **(C, D)**, presence of perineural invasion (PNI) **(E, F)**, and presence of lymphovascular invasion (LVI) **(G, H)**. HR, hazard ratio; CI, 95% confidence interval.

Baseline characteristics for the OSCC patients are summarized in **Table 1**. Fifty eight percent of the patients were male and 42% were female. Female patients were diagnosed at an older average age then male patients (66 versus 58 years respectively, p=0.002, **Table 1**), although there were no significant differences in survival outcomes (OS or PFS) between sexes (**Supplemental Figures 1A, B**). Patients 60 years and older demonstrated significantly worse OS (but not PFS) compared to patients under 60 years (**Figures 1A, B**). Active smokers comprised 44% of patients, with 35% having never smoked, 9% which quit smoking for less than 10 years, and 12% which quit smoking for more than 10 years (**Table 1**). There was no difference in OS between active smokers, never smokers and patients that quit (p=0.2, **Figure 1C**), however significant differences were observed in PFS where never smokers had improved PFS compares to active and patients that quit smoking (p=0.04, **Figure 1D**). Of the oral cavity disease sites, tongue was the most common disease location (38%, **Table 1**) and there was a trend toward differences in OS (p=0.07) but not PFS (p=0.38) by tumor site (**Supplemental Figures 1C, D**). Patients with T3/T4 tumors represented 41% of the patient cohort (**Table 1**) and showed a trend toward worse OS compared to T1 and T2 tumors (p=0.06) but no differences in PFS (**Supplemental Figures 1E, F**). The majority of patients presented with no lymph node metastasis (N0, 51%, **Table 1**) and demonstrated significantly more favorable OS (but not PFS) compared to patients that presented with N1 or N2/N3 disease (**Figures 2A, B**). Patients with poorly differentiated tumors represented 25% of the patient cohort (**Table 1**) and were associated with a trend toward worse OS (p=0.08, **Figure 2C**) and significantly worse PFS (p=0.04, **Figure 2D**) compared to well and moderately differentiated tumors. Perineural (PNI), lymphovascular (LVI) and bone invasion (BI) were observed in 50%, 37% and 30% of tumors respectively (**Table 1**). Patients presenting with PNI demonstrated significantly worse OS (p=0.004, **Figure 2E**) and PFS (p=0.04, **Figure 2F**) compared to patients with no PNI. Patients presenting with LVI demonstrated significantly worse OS (p=0.004, **Figure 2G**) and a trend toward worse PFS (p=0.06, **Figure 2H**) compared to patients with no LVI. BI status did not affect OS nor PFS in the patient cohort (**Supplemental Figures 1G, H**). The average follow-up time was 107 ± 37 months. Of the 146 cases, 48 patients survived 5 years after diagnoses.

**Table 1:** Clinicopathological features of patients based on EGFR status.

Characteristics	Total Patients (n)	EGFR Status (n=143)		p-value
		EGFR+	EGFR-	
Number of evaluations	146	59	84	
**Sex **				
Male	85 (58.22%)	34 (57.63%)	48 (57.14%)	0.91
Female	61 (41.78%)	25 (42.37%)	36 (42.86%)	
Average age [ ± stdev]*				
Male	58.16 [11.05]	58.03 [13.03]	58.33 [9.94]	0.95
Female	66.41 [17.36]	67.04 [16.31]	65.97 [18.29]	
**Smoking History **				
Active smoker	64 (44.14%)	24 (40.68%)	37 (44.58%)	0.32
Never smoker	50 (34.48%)	22 (37.29%)	28 (33.73%)	
Quit < 10 Years	13 (8.97%)	3 (5.08%)	10 (12.05%)	
Quit > 10 Years	18 (12.41%)	10 (16.95%)	8 (9.64%)	
N/A	1			
**Tumor Site **				
Alveolar	24 (16.44%)	9 (15.25%)	14 (16.67%)	0.41
Floor of mouth	32 (21.92%)	11 (18.64%)	21 (25%)	
Tongue	55 (37.67%)	27 (45.76%)	27 (32.14%)	
Other	35 (23.97%)	12 (20.34%)	22 (26.19%)	
**T Stage**				
T1	43 (29.45%)	22 (37.29%)	21 (25%)	0.27
T2	43 (29.45%)	16 (27.12%)	25 (29.76%)	
T3/T4	60 (41.10)	21 (35.59%)	38 (45.24)	
**N Stage**				
N0	74 (50.68%)	27 (45.76%)	46 (54.76%)	0.16
N1/2a	29 (19.86%)	16 (27.12%)	12 (14.29%)	
N2b/2c/3	43 (29.45%)	16 (27.12%)	26 (30.95%)	
**Differentiation**				
Well	15 (10.49%)	3 (5.26%)	12 (14.46%)	0.22
Moderate	92 (64.34%)	38 (66.67%)	51 (61.45%)	
Poor	36 (25.17%)	16 (28.07%)	20 (24.1%)	
N/A	3			
**Perineural invasion**				
Yes	73 (50.34%)	35 (59.32%)	38 (45.24%)	0.1
No	72 (49.66%)	24 (40.68%)	46 (54.76%)	
N/A	1			
**Lymphovascular invasion**				
Yes	54 (37.24%)	25 (42.37%)	29 (34.52%)	0.34
No	91 (62.76%)	34 (57.63%)	55 (65.48%)	
N/A	1			
**Bone invasion**				
Yes	44 (30.34%)	15 (25.42%)	29 (34.52%)	0.25
No	101 (69.66%)	44 (74.58%)	55 (65.48%)	
N/A	1			
**Local recurrence**				
Yes	45 (30.82%)	20 (33.90%)	25 (29.76%)	0.6
No	101 (69.18%)	39 (66.10%)	59 (70.24%)	

*Average age at diagnosis

### Prognostic Impact by EGFR Expression

**Figure 3:**
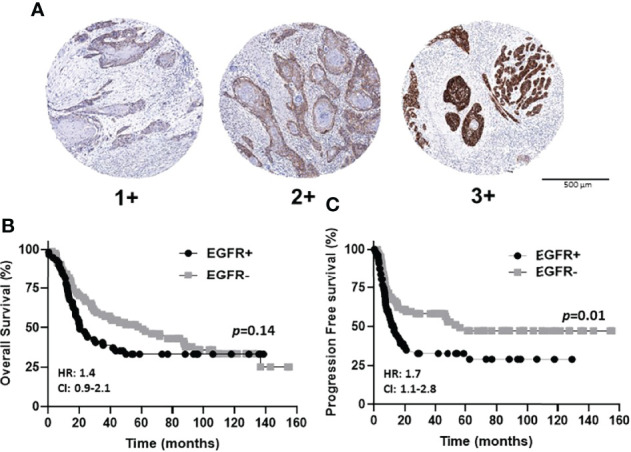
EGFR immunostaining and expression scores in OSCCs. **(A)**: Shown are images of low [1+], moderate [2+], and strong [3+] EGFR expression scores. **(B, C)**: Shown are Kaplan-Meier estimates of the overall survival **(B)** and progression free survival **(C)** of EGFR+ (2+ and 3+) and EGFR- (0 and 1+) patients. HR, hazard ratio; CI, 95% confidence interval.

The prognostic value of EGFR expression in the OSCC cases represented in the TMA was initially evaluated. Examples of EGFR expression scores are shown in **Figure 3A**. EGFR+ expression represents tumors with strong (score of 3) and moderate (score of 2) expression, while EGFR- expression represents low (score of 1) and no (score of 0) expression. Patient clinicopathological characteristics based on EGFR status (EGFR+ vs EGFR-) are shown in **Table 1** where there were no significant associations observed. There were also no differences observed in OS (*p*=0.14) according to EGFR+ and EGFR- expression (**Figure 3B**). However significant differences were observed in PFS (*p*=0.01) with higher EGFR expression (EGFR+) being associated with worse PFS (**Figure 3C**). Similar results with OS and PFS were obtained when the prognostic value of each EGFR expression score (3, 2, 1 and 0) was previously evaluated shown here (58). However, the combined (3 + 2 [EGFR+], 1 + 0 [EGFR-]) expression scores were utilized for the remainder of the study to maintain sufficient case numbers to further analyze T cell subsets in these EGFR expression groups. These results support prior reports that EGFR expression is a strong predictor for PFS but not OS in OSCC patients (27, 59).

### Prognostic Impact of CD3+ Tumor-Infiltrating Lymphocyte Marker Expression

**Figure 4:**
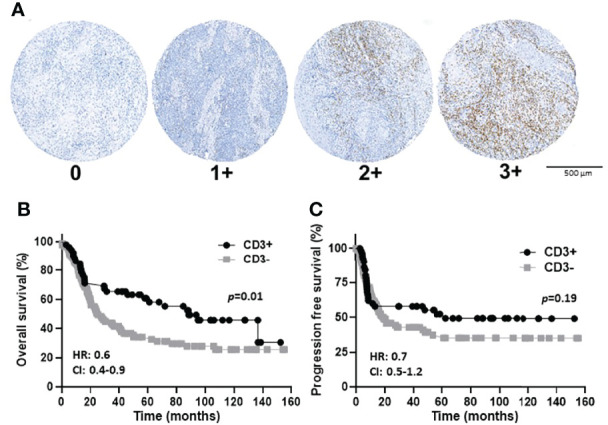
CD3 immunostaining and expression scores in OSCCs. **(A)**: Shown are images of no [0], low [1+], moderate [2+], and strong [3+] CD3 expression scores. **(B, C)**: Shown are Kaplan-Meier estimates of the overall survival **(B)** and progression free survival **(C)** of CD3+ (2+ and 3+) and CD3- (0 and 1+) patients. HR, hazard ratio; CI: 95% confidence interval.

**Figure 5:**
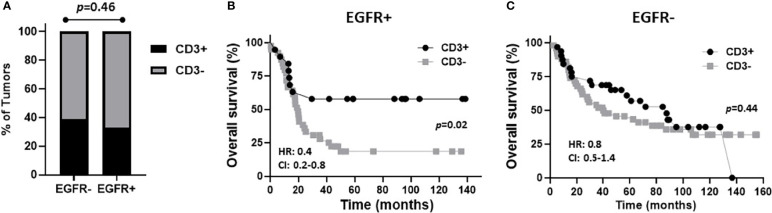
Prognostic impact of combined EGFR and CD3 expression in OSCCs. **(A)**: Shown are percentages of tumors with CD3+ and CD3- expression based on EGFR status. **(B, C)**: Shown are Kaplan-Meier estimates of overall survival of CD3+ and CD3- patients based on EGFR+ **(B)** or EGFR- **(C)** tumor expression. HR, hazard ratio; CI: 95% confidence interval.

The prognostic value of TIL expression was initially assessed by pan-T cell (CD3) expression. Images of no [0], low [1+], moderate [2+], and strong [3+] CD3 expression scores are shown in **Figure 4A**. CD3 expression scores were combined in a manner identical to EGFR above where combined 3 and 2 scores represent CD3+ and combined 1 and 0 scores represented CD3-. CD3+ expression was associated with tongue tumors compared to other disease sites (p=0.018), and also associated with cases presenting with no lymph node metastasis (N0) compared to N1 and N2/3 cases (p=0.008) (**Table 2**). Additionally, CD3+ OSCCs were associated with favorable OS compared to CD3- tumors (p=0.01, **Figure 4B**). CD3 expression did not impact PFS (p=0.19, **Figure 4C**). These results suggest that CD3+ TILs may suppress lymph node metastasis and thus survival outcomes. The prognostic value of CD3 was next evaluated in EGFR+ and EGFR- OSCC patients. There was no difference in CD3 expression between EGFR+ and EGFR- tumors (**Figure 5A**). However, EGFR+/CD3+ expression was most frequently observed in younger males (p=0.01) and associated with less lymph node metastasis (p=0.046) compared to EGFR+/CD3- patients (**Table 3**). Patients with CD3+ OSCCs were associated with significantly higher OS (p=0.02, cox ph = 0.0097) compared to CD3- tumors, but only in EGFR+ patients (**Figure 5B**) and not EGFR- patients (**Figure 5C**). There were no differences in PFS observed with any of the combined EGFR/CD3 expression scores (**Supplemental Figures 2A, B**).

**Table 2:** Clinicopathological features of patients based on T Cell marker status.

Characteristics	Total cases(n)	CD3 Status		p-value	Total cases(n)	CD4 Status		p-value	Total cases(n)	CD8 Status		p-value
		CD3+	CD3-			CD4+	CD4-			CD8+	CD8-	
**Number of evaluations **	141	52 (36.88%)	89 (63.12%)		143	49 (34.27%)	94 (65.73%)		139	90 (64.75%)	49 (35.25%)	
**Sex**												
Male	80	33 (63.46%)	47 (52.81%)	0.22	83	30 (61.22%)	53 (38.78%)	0.58	79	56 (62.22%)	23 (46.94%)	0.08
Female	61	19 (36.54%)	42 (47.19%)		60	19 (56.38%)	41 (43.62%)		60	34 (37.78%)	26 (53.06%)	
Average age* **[ ± stdev]**												
Male	80	54 [10.79]	61 [10.21]	0.86	83	57 [10.09]	58.87 [11.79]	0.97	79	57.87 [11.29]	56.32 [11.61]	0.80
Female	61	63 [20.12]	68 [10.92]		60	64.47 [23.18]	68.07 [13.47]		60	66.2 [17.44]	68.23 [13.61]	
**Smoking History **												
Active smoker	61	22 (42.31%)	39 (44.32%)	0.72	62	15 (31.25%)	47 (50%)	0.09	60	34 (38.20%)	26 (53.06%)	0.34
Never smoker	50	17 (32.69%)	33 (37.5%)		50	22 (45.83%)	28 (29.79%)		47	32 (35.96%)	15 (30.61%)	
Quit<10 Years	13	9 (17.31%)	4 (4.55%)		12	6 (1.25%)	(6.38%)		13	9 (10.11%)	4 (8.16%)	
Quit>10 Years	16	4 (7.69%)	12 (13.64%)		18	5 (1.04%)	13 (13.83%)		18	14 (15.73%)	4 (8.16%)	
N/A	1				1				1			
**Tumor Site**												
Alveolar	23	7 (13.46%)	16 (17.98%)	0.018	23	7 (14.29%)	16 (17.02%)	0.004	21	16 (17.78%)	5 (10.20%)	0.08
Floor of mouth	32	6 (11.54%)	26 (29.21%)		32	3 (6.12%)	29 (30.85%)		29	14 (15.56%)	15 (30.61%)	
Tongue	52	27 (51.92%)	25 (28.09%)		54	25 (51.02%)	29 (30.85%)		55	40 (44.44%)	15 (30.61%)	
Other	34	12 (23.08%)	22 (24.72%)		34	14 (28.57%)	20 (21.28%)		34	20 (22.22%)	14 (28.57%)	
**T Stage**												
T1	42	20 (38.46%)	22 (24.72%	0.06	43	19 (38.78%)	24 (25.53%)	0.13	43	30 (33.33%)	13 (26.53%)	0.49
T2	41	17 (32.69%)	24 (26.97%)		41	15 (30.61%)	26 (27.66%)		40	27 (30%)	13 (26.53%)	
T3/T4	58	15 (28.85%)	43 (48.31%)		59	15 (30.61%)	44 (46.81%)		56	33 (36.67%)	23 (46.94%)	
**N Stage**											
N0	72	34 (65.38%)	38 (42.70%)	0.008	73	33 (67.35%)	40 (42.55%)	0.019	71	50 (55.56%)	21 (42.86%)	0.32
N1/2a	28	4 (7.69%)	24 (26.97%)		28	6 (12.24%)	22 (23.40%)		27	15 (16.67%)	12 (24.49%)	
N2b/2c/3	41	14 (26.92%)	27 (30.34%)		42	10 (20.41%)	32 (34.04%)		41	25 (27.78%)	16 (32.65%)	
**Differentiation**												
Well	15	8 (15.38%)	7 (8.14%)	0.41	15	9 (18.75%)	6 (6.52%)	0.06	15	11 (12.5%)	4 (8.33%)	0.69
Moderate	89	32 (61.54%)	57 (66.28%)		89	26 (54.17%)	63 (68.48%)		85	53 (60.23%)	32 (66.67%)	
Poor	34	12 (23.08%)	22 (25.58%)		36	13 (27.08%)	23 (25%)		36	24 (27.27%)	12 (25%)	
N/A	3				3				3			
**Perineural invasion**												
Yes	73	24 (46.15%)	49 (55.06%)	0.31	72	20 (40.82%)	52 (55.32%)	0.1	73	44 (48.89%)	29 (59.18%)	0.25
No	68	28 (53.85%)	40 (44.94%)		71	29 (59.18%)	42 (44.68%)		66	46 (51.11%)	20 (40.82%)	
**Lymphovascular** **invasion**												
Yes	54	18 (34.62%)	36 (40.45%)	0.49	54	11 (22.45%)	43 (45.74%)	0.006	53	32 (35.56%)	21 (42.86%)	0.4
No	87	34 (65.38%)	53 (59.55%)		89	38 (77.55%)	51 (54.26%)		86	58 (64.44%)	28 (57.14%)	
**Bone** **invasion**												
Yes	44	13 (25%)	31 (34.83%)	0.22	44	10 (20.41%)	34 (36.17%)	0.052	41	27 (30%)	14 (28.57%)	0.86
No	97	39 (75%)	58 (65.17%		99	39 (79.59%)	60 (63.83%)		98	63 (70%)	35 (71.43%)	
**Local recurrence**												
Yes	44	15 (28.85%)	29 (32.58%)	0.64	45	18 (36.74%)	27 (28.72%)	0.33	44	29 (32.22%)	15 (30.61%)	0.85
No	97	37 (71.15%)	60 (67.42%)		98	31 (63.27%)	67 (71.28%)		95	61 (67.78%)	34 (69.39%)	

*Average age at diagnosis.

**Table 3:** Clinicopathological features of patients based on EGFR/CD3 status.

Characteristics	Total cases	EGFR+		p-value	Total cases	EGFR-		p-value
		CD3+	CD3-			CD3+	CD3-	
**Number of Cases**	58	19 (32.76%)	39 (67.24%)		82	32 (39.02%)	50 (60.98%)	
**Sex**								
Male	33	13 (68.42%)	20 (51.28%)	0.2	46	19 (59.38%)	27 (54%)	0.63
Female	25	6 (31.58%)	19 (48.72%)		36	13 (40.63%)	23 (46%)	
Average Age [ ± stdev]*								
Male	33	50.46 [10.81]	62.4 [12.47]	0.01	46	56.79 [10.57]	59.52 [8.20]	0.22
Female	25	62 [25.81]	68.63 [12.58]		36	63.92 [18.12]	67.13 [18.68]	
**Smoking History**								
Active smoker	24	8 (42.11%)	16 (41.02%)	0.31	36	13 (40.63%)	23 (46%)	0.19
Never smoker	22	8 (42.11%)	14 (35.90%)		28	9 (28.13%)	19 (38%)	
Quit < 10 Years	3	2 (10.53%)	1 (2.56%)		10	7 (21.88%)	3 (6%)	
Quit > 10 Years	9	1 (5.26%)	8 (20.51%)		7	3 (9.38%)	4 (8%)	
N/A	–				1			
**Tumor Site**								
Alveolar	9	3 (15.79%)	6 (15.38%)	0.016	14	4 (12.5%)	10 (20%)	0.1
Floor of mouth	11	1 (5.26%)	10 (25.64%)		21	5 (15.63%)	16 (32%)	
Tongue	26	12 (63.16%)	14 (35.90%)		25	14 (43.75%)	11 (22%)	
Other	12	3 (15.79%)	9 (23.08%)		22	9 (28.13%)	13 (26%)	
**T Stage**								
T1	22	8 (42.11%)	14 (35.90%)	0.54	20	12 (37.50%)	8 (16%)	0.05
T2	15	6 (31.58%)	9 (23.08%)		25	10 (31.25%)	15 (30%)	
T3/T4	21	5 (26.31%)	16 (41.02%)		37	10 (31.25%)	27 (54%)	
**N Stage**								
N0	27	13 (68.42%)	14 (35.90%)	0.05**	44	20 (62.50%)	24 (48%)	0.19
N1/2a	16	2 (10.53%)	14 (35.90%)		12	2 (6.25%)	10 (20%)	
N2b/2c/3	15	4 (21.05%)	11 (28.20%)		26	10 (31.25%)	16 (32%)	
**Differentiation**								
Well	3	2 (10.53%)	1 (2.56%)	0.41	12	6 (18.75%)	6 (12%)	0.65
Moderate	38	13 (68.42%)	25 (64.10%)		50	18 (56.25%)	32 (64%)	
Poor	15	4 (21.05%)	11 (28.20%)		19	8 (25%)	11 (22%)	
N/A	2				1			
**Perineural invasion**								
Yes	35	12 (63.16%)	23 (58.97%)	0.76	38	12 (37.50%)	26 (52%)	0.19
No	23	7 (36.84%)	16 (41.03%)		44	20 (62.50%)	24 (48%)	
**Lymphovascular invasion**								
Yes	25	7 (36.84%)	18 (46.15%)	0.50	29	11 (34.38%)	18 (36%)	0.88
No	33	12 (63.16%)	21 (53.85%)		53	21 (65.63%)	32 (64%)	
**Bone invasion**								
Yes	15	4 (21.05%)	11 (28.20%)	0.56	29	9 (28.13%)	20 (40%)	0.27
No	44	15 (78.95%)	28 (71.80%)		53	23 (71.88%)	30 (60%)	
**Local recurrence**								
Yes	20	6 (31.58%)	14 (35.90%)	0.75	24	9 (28.13%)	15 (30%)	0.86
No	38	13 (68.42%)	25 (64.10%)		58	23 (71.88%)	35 (70%)	

*Average age at diagnosis. **p = 0.046.

### Prognostic Impact of CD4+ and CD8+ Tumor-Infiltrating Lymphocyte Marker Expression

**Figure 6:**
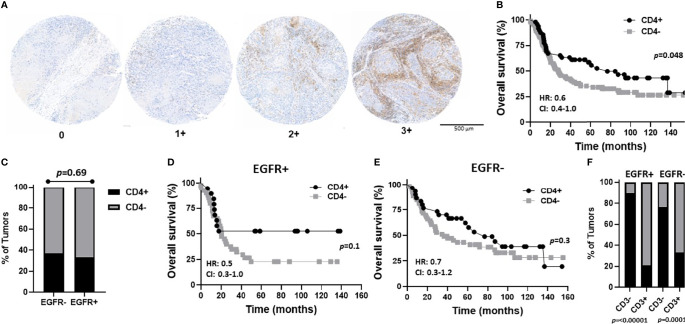
Prognostic impact of combined EGFR and CD4 expression in OSCCs. **(A)** Shown are images of no [0], low [1+], moderate [2+], and strong [3+] CD4 expression scores. **(B)** Kaplan-Meier estimates of the overall survival of CD4+ (2+, 3+) and CD4- (0, 1+) patients. **(C)** Percentages of tumors with CD4+ and CD4- expression based on EGFR status. **(D, E)** Kaplan-Meier estimates of overall survival of CD4+ and CD4- patients based on EGFR+ **(D)** or EGFR- **(E)** tumor expression. **(F)** Percentages of tumors with CD4+ and CD4- expression based on EGFR and CD3 status. HR, hazard ratio; CI, 95% confidence interval.

In order to determine what type of TIL represented the CD3 expression observed in the EGFR+/CD3+ patients, CD4 and CD8 expression was assessed in each OSCC case. CD4 expression scores and images are shown in **Figure 6A** and were combined as carried out for EGFR and CD3 where 3 and 2 scores represented CD4+ and combined 1 and 0 scores represented CD4-. Patient clinicopathological characteristics based on CD4 expression are shown in **Table 2**. CD4+ expression was associated with tongue tumors, no lymph node metastasis (N0, p=0.019) and no LVI (p=0.006, **Table 2**). CD4- expression were associated with floor of mouth tumors (p=0.004, **Table 2**). CD4- OSCCs were associated with significantly lower OS compared to CD4+ OSCCs (p=0.048, **Figure 6B**) and there was no impact on PFS (**Supplemental Figure 3A**). When combined EGFR and CD4 expression was analyzed, it was found that EGFR+/CD4+ expression was associated with never smokers (p=0.03), tongue tumor disease site (p=0.03), T1 tumors (p=0.02), N0 tumors (p=0.002), no LVI (p=0.02) and no BI (p=0.01) (**Table 4**). There was no difference in CD4 expression between EGFR+ and EGFR- tumors (p=0.69, **Figure 6C**) and there were no differences observed in OS (**Figures 6D, E**) or PFS (**Supplemental Figures 3B, C**) with any of the combined EGFR/CD4 expression scores. Patients that were CD3+ also were more likely to be CD4+, but EGFR status had no influence on this observation (**Figure 6F**). These results suggest that combined EGFR+/CD4+ expression is associated with favorable clinicopathological features for survival.

**Table 4:** Clinicopathological features of patients based on EGFR/CD4 status.

**Characteristics**	**Total cases**	**EGFR+**		**p-value**	**Total cases**	**EGFR-**		**p-value**
		**CD4+**	**CD4-**			**CD4+**	**CD4-**	
**Number of Cases**	59	19 (32.2%)	40 (67.8%)		83	30 (36.14%)	53 (63.86%)	
**Sex**								
Male	34	11 (57.89%)	23 (57.5%)	0.98	48	19 (63.33%)	29 (54.72%)	0.45
Female	25	8 (42.11%)	17 (42.5%)		35	11 (36.67%)	24 (45.28%)	
Average Age [ ± stdev]*								
Male	34	52.27 ± 10.69	60.78 ± 13.35		48	59.74 ± 8.88	57.41 ± 10.63	
Female	25	63.75 ± 23.44	68.59 ± 12.26		35	65 ± 24.11	67.71 ± 14.52	
**Smoking History**								
Active smoker	24	5 (26.32%)	19 (47.5%)	0.03	34	10 (34.48%)	27 (50.94%)	0.46
Never smoker	22	10 (52.63%)	12 (30%)		28	12 (41.38%)	16 (30.19%)	
Quit < 10 Years	3	3 (15.79%)	0 (0%)		9	3 (10.34%)	6 (11.32%)	
Quit > 10 Years	10	1 (5.26%)	9 (22.5%)		8	4 (13.79%)	4 (7.55%)	
N/A	–				1			
**Tumor Site**								
Alveolar	9	3 (15.79%)	6 (15%)	0.03	14	4 (13.33%)	10 (18.87%)	0.04
Floor of mouth	11	0 (0%)	11 (27.5%)		21	3 (10%)	18 (33.96%)	
Tongue	27	14 (73.68%)	13 (32.5%)		26	11 (36.67%)	15 (28.30%)	
Other	12	2 (10.53%)	10 (25%)		22	12 (40%)	10 (18.87%)	
**T Stage**								
T1	22	10 (52.63%)	12 (30%)	0.02	21	9 (30%)	12 (22.64%)	0.76
T2	16	7 (36.84%)	9 (22.5%)		24	8 (26.67%)	16 (30.19%)	
T3/T4	21	2 (10.53%)	19 (47.5%)		38	13 (43.33%)	25 (47.17%)	
**N Stage**								
N0	27	15 (78.94%)	12 (30%)	0.002	45	18 (60%)	27 (50.94%)	0.72
N1/2a	16	2 (10.53%)	14 (35%)		12	4 (13.33%)	8 (15.09%)	
N2b/2c/3	16	2 (10.53%)	14 (35%)		26	8 (26.67%)	18 (33.96%)	
**Differentiation**								
Well	3	2 (11.11%)	1 (2.56%)	0.36	12	7 (23.33%)	5 (9.62%)	0.1
Moderate	38	12 (66.67%)	26 (66.67%)		50	14 (46.67%)	36 (69.23%)	
Poor	16	4 (22.22%)	12 (30.77%)		20	9 (30%)	11 (21.15%)	
N/A	2				1			
**Perineural invasion**								
Yes	35	9 (47.37%)	26 (65%)	0.20	37	11 (36.67%)	26 (49.06%)	0.28
No	24	10 (52.63%)	14 (35%)		46	19 (63.33%)	27 (50.94%)	
**Lymphovascular invasion**								
Yes	25	4 (21.05%)	21 (52.5%)	0.02	29	7 (23.33%)	22 (41.51%)	0.1
No	34	15 (78.95%)	19 (47.5%)		54	23 (76.67%)	31 (58.49%)	
**Bone invasion**								
Yes	15	1 (5.26%)	14 (35%)	0.01	29	9 (30%)	20 (37.74%)	0.48
No	44	18 (94.74%)	26 (65%)		54	21 (70%)	33 (62.26%)	
**Local recurrence**								
Yes	20	8 (42.11%)	12 (30%)	0.36	25	10 (33.33%)	15 (28.30%)	0.63
No	39	11 (57.89%)	28 (70%)		58	20 (66.67%)	38 (71.70%)	

*Average age at diagnosis.

**Figure 7:**
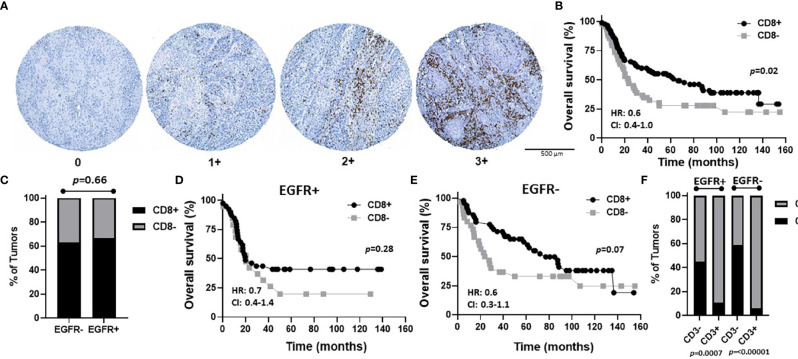
Prognostic impact of combined EGFR and CD8 expression in OSCCs. **(A)** Shown are images of no [0], low [1+], moderate [2+], and strong [3+] CD8 expression scores. **(B)** Kaplan-Meier estimates of the overall survival of CD8+ (2+, 3+, 1+) and CD8- (0) patients. **(C)** Percentages of tumors with CD8+ and CD8- expression based on EGFR status. **(D, E)** Kaplan-Meier estimates of overall survival of CD8+ and CD8- patients based on EGFR+ **(D)** or EGFR- **(E)** tumor expression. **(F)** Percentages of tumors with CD8+ and CD8- expression based on EGFR and CD3 status. HR, hazard ratio; CI, 95% confidence interval.

To assess CD8 expression, scores of 0-3 were again assigned as shown in **Figure 7A**. However, due to the low number of patients with CD8 scores of 3 (n=2) and 2 (n=11), we used scores of 3, 2, and 1 to represent CD8+ tumors (n=90) and a score of 0 to represent CD8- tumors (n=49) in order to have sufficient numbers for group comparisons and further subset analyses. Patient clinicopathological characteristics based on CD8 expression are included in **Table 2** and there was no significant association between CD8 expression and any of the characteristics analyzed. However, CD8+ OSCCs were associated with significantly more favorable OS compared to CD8- OSCCs (p=0.02, **Figure 7B**) and there was no impact on PFS (**Supplemental Figure 3D**). When combined EGFR and CD8 expression was analyzed, EGFR+/CD8+ was associated with tumors in male patients but no other associations with clinicopathological characteristics were observed (**Table 5**). There were no differences in CD8 expression between EGFR+ and EGFR- tumors (p=0.66, **Figure 7C**). There were also no differences observed in OS (**Figures 7D, E**) or PFS (**Supplemental Figures 3E, F**) with any of the combined EGFR/CD8 expression scores. Notably, EGFR-/CD8+ expression showed a trend (p=0.07) toward association with favorable OS compared with EGFR-/CD8- expression when analyzed by log-rank test (**Figure 7E**), but showed significance (p=0.01) when the Gehan-Breslow-Wilcoxon test was used which gives more weight to deaths that occur at early time points. As shown with CD4+ (**Figure 6F**), patients that were CD3+ were also more likely to be CD8+ but EGFR status had no influence on this observation (**Figure 7F**).

**Table 5:** Clinicopathological features of patients based on EGFR/CD8 status.

Characteristics	Total cases	EGFR+		p-value	Total cases	EGFR-		p-value
		CD8+	CD8-			CD8+	CD8-	
**Number of Cases**	58	39 (67.24%)	19 (32.76%)		80	50 (62.5%)	30 (37.5%)	
**Sex**								
Male	33	26 (66.67%)	7 (36.84%)	0.03	45	29 (58%)	16 (53.33%)	0.68
Female	25	13 (33.33%)	12 (63.16%)		35	21 (42%)	14 (46.67%)	
Average Age [± stdev]*								
Male	33	59.08 [13.21]	53.57 [13.24]	0.06	45	58 [9.72]	57.63 [10.66]	0.18
Female	25	63 [19.28]	71.42 [11.60]		35	65.67 [20.74]	65.5 [15]	
**Smoking History**								
Active smoker	23	13 (33.33%)	10 (52.63%)	0.66	36	20 (40.82%)	16 (53.33%)	0.41
Never smoker	22	16 (41.03%)	6 (31.58%)		25	16 (32.65%)	9 (30%)	
Quit < 10 Years	3	3 (7.69%)	0 (0%)		10	6 (12.24%)	4 (13.33%)	
Quit > 10 Years	10	7 (17.95%)	3 (15.79%)		8	7 (14.29%)	1 (3.33%)	
N/A					1			
**Tumor Site**								
Alveolar	9	7 (17.95%)	2 (10.53%)	0.05	12	9 (18%)	3 (10%)	0.40
Floor of mouth	10	5 (12.82%)	5 (26.32%)		19	9 (18%)	10 (33.33%)	
Tongue	27	22 (56.41%)	5 (26.32%)		27	17 (34%)	10 (33.33%)	
Other	12	5 (12.82%)	7 (36.84%)		22	15 (30%)	7 (23.33%)	
**T Stage**								
T1	22	15 (38.46%)	7 (36.84%)	0.63	21	15 (30%)	6 (20%)	0.60
T2	16	12 (30.77%)	4 (21.05%)		23	14 (28%)	9 (30%)	
T3/T4	20	12 (30.77%)	8 (42.11%)		36	21 (42%)	15 (50%)	
**N Stage**								
N0	26	20 (51.28%)	6 (31.58%)	0.34	44	29 (58%)	15 (50%)	0.75
N1/2a	16	9 (23.08%)	7 (36.84%)		11	6 (12%)	5 (16,67%)	
N2b/2c/3	16	10 (25.64%)	6 (31.58%)		25	15 (30%)	10 (33.33%)	
**Differentiation**								
Well	3	3 (7.89%)	0 (0%)	0.28	12	8 (16.32%)	4 (13.33%)	0.74
Moderate	37	22 (57.89%)	15 (83.33%)		47	30 (61.22%)	17 (56.67%)	
Poor	16	13 (34.21%)	3 (16.67%)		20	11 (22.45%)	9 (30%)	
N/A	2				1			
**Perineural invasion**								
Yes	35	23 (58.97%)	12 (63.16%)	0.76	38	21 (42%)	17 (56.67%)	0.2
No	23	16 (41.03%)	7 (36.84%)		42	29 (58%)	13 (43.33%)	
**Lymphovascular invasion**								
Yes	25	15 (38.46%)	10 (52.63%)	0.31	28	17 (34%)	11 (36.67%)	0.81
No	33	24 (61.54%)	9 (47.37%)		52	33 (66%)	19 (63.33%)	
**Bone invasion**								
Yes	14	9 (23.08%)	5 (26.32%)	0.79	27	18 (36%)	9 (30%)	0.58
No	44	30 (76.92%)	14 (73.68%)		53	32 (64%)	21 (70%)	
**Local recurrence**								
Yes	19	13 (33.33%)	6 (31.58%)	0.89	25	16 (32%)	9 (30%)	0.85
No	39	26 (66.67%)	13 (68.42%)		55	34 (68%)	21 (70%)	

*Average age at diagnosis.

Altogether, these results demonstrate that combined EGFR+/CD3+ expression identified a subset of OSCC patients with favorable prognosis compared to other biomarker profiles which may be attributed to a lack of lymph node metastasis.

## Discussion

The data shown here suggests that CD3 positivity is a prognostic biomarker for OSCC patients (**Figure 4B**) but only for EGFR+ tumors (**Figure 5B**). Hence, a combined EGFR/CD3 expression profile could potentially be used to make important treatment related decisions for EGFR+ patients that typically have an increased likelihood of tumor recurrence and progression (**Figure 3C**). For example, agents that re-direct T cells toward EGFR+ tumors such as EGFR-CD3 bispecific antibodies (60), can be administered to EGFR+/CD3- patients. Alternatively, agents that enhance T cell activity such as checkpoint inhibitors, can be administered to EGFR+/CD3+ patients.

Unfortunately, EGFR expression is not routinely tested for in the clinical setting for OSCC due to (1) EGFR expression not necessarily correlating with EGFR activity, (2) EGFR expression not predicting response to EGFR inhibitors, and (3) cetuximab (CTX, EGFR inhibitor) being administered to patients despite EGFR expression (24–26). This would also explain why CTX-based therapy has only modest impact on OSCC patients and in most cases is being replaced with immunotherapy (61). However, these therapy-related issues related to EGFR do not negate the important role of EGFR signaling in tumor growth and aggressiveness or the clear role of EGFR as a prognostic biomarker for PFS (24, 7).

As immunotherapy has now moved to the forefront of cancer therapy, immune biomarkers are of great interest. The expression of T cell markers can give an idea of the status of the immune microenvironment and possibly host immune responses (41). Increased expression and/or density of CD3 (pan-T cells) and CD8 (cytotoxic T cells) have previously shown associations with OS in a variety of disease models (37–40) which we also confirmed in the present studies (**Figures 4B**, **7B**). The prognostic role of CD4 in the research literature is questionable (39, 41, 62, 63), although our studies found a significant prognostic role of CD4 in OS (**Figure 6B**). Among the OSCC locations, floor of the mouth tumors were more likely to CD4- and tumors of the tongue were more likely to express higher levels of T cell markers (especially CD3 and CD4) compared to the other oral cavity subsites. CD4+ expression was also associated with an absence of LVI (**Table 2**). We are unclear of why these TIL differences in oral cavity subsites occur, but we can reason that due to its location and function in the oral cavity, the tongue is constantly challenged by antigens from food and air. It is possible that the tongue (and tumors derived from the tongue) are T cell rich due to the abundance of T-cells residing in the mucosa that normally control mucosal immunity and tolerance (64). Nevertheless, our findings support prior work which found that most of tongue tumors are “TIL-high” (65, 66), the TILs were associated with absence of LVI (67) and were likely CD4+ T cells (68).

In this study we probed if EGFR could be combined with a TIL marker with the rationale that combining independent prognostic markers may increase the accuracy or reliability of assessing survival outcomes. EGFR expression would provide information about tumor aggressiveness while TIL marker(s) expression would give some input regarding the tumor immune microenvironment and overall prognosis. We found that combined EGFR/CD3 expression provided important prognostic information where increased CD3 expression was associated with improved OS in EGFR+ patients (**Figure 5B**). These results were surprising since we initially proposed based off individual EGFR (**Figure 3B**) and CD3 (**Figure 4B**) survival curves, that an EGFR-/CD3+ expression profile would be associated with the most favorable survival outcomes. However, CD3 expression provided no prognostic value in EGFR- tumors (**Figure 5C**). Perhaps since EGFR is a self-antigen, increased EGFR expression may trigger an EGFR-specific T cell response which would explain why EGFR+/CD3+ patients have a favorable OS. In support of this idea, previous work has shown that EGFR expressed on HNSCC cells induces a specific immune response *in vivo* and that higher EGFR expression was associated with increased circulation of EGFR-specific CD8+ T cells (69).

The majority of patients with high CD3 expression were high for CD4 (**Figure 6F**) and CD8 (**Figure 7F**) expression regardless of EGFR expression. Therefore, it is difficult to assess if increased CD4+ and/or CD8+ T cell activity is responsible for the favorable OS observed in EGFR+/CD3+ patients. Interestingly, combined EGFR+/CD4+ expression was associated with a variety of characteristics associated for favorable survival such as history of not smoking, T1 and N0 stage, and absence of LVI and BI (**Table 4**). However, EGFR+/CD4+ expression was not associated with OS or PFS despite these findings (**Figures 6D,E**). Future work will pursue these observations and further determine the expression of subsets of CD4+ T cells in OSCCs combined with EGFR. We expected to observe superior survival outcomes in EGFR-/CD8+ patients since EGFR- and CD8+ expression as separate entities are associated with more favorable outcomes compared to EGFR+ and CD8- respectively. In support of this, we found a strong trend (p=0.07) between combined EGFR-/CD8+ expression and survival outcomes (**Figure 7E**). The sole explanation for the favorable OS observed in EGFR+/CD3+ patients was the significant association with decreased lymph node metastasis (**Table 2**). This finding is quite contradictory since increased EGFR is associated with increased lymph node metastasis (70) but increased CD3 expression has been associated with decreased lymph node metastasis (38). It is unclear how the presence of CD3+ T cells overrides the tumor promoting effects of EGFR.

Limitations of this study are that 1 – the work was conducted in a retrospective fashion; 2 - all cases were from a single institution; 3 – TMAs allow for only a limited area of tumor (and stroma) components for evaluation; 4 – TMA consisted of a relatively small sample size; and 5 – lack of HPV+ OSCCs in the patient cohort. Although HPV OSCCs are rare, there is evidence of HPV’s role in predicting patient prognosis (71) and there may be a benefit to including HPV+ cases in our future studies. Lastly, it is acknowledged that the expression of inhibitory checkpoints including programmed cell death-ligand 1 (PD-L1) and programmed cell death-protein 1 (PD1) regulate T-cell response (72, 73). Future studies will determine if immune checkpoint marker expression would explain some of the contradictory results observed in the present studies.

Overall, our findings suggest that the expression of CD3 was superior to CD4 and CD8 at enhancing the prognostic value of EGFR in OSCC patients. This work warrants further investigation of EGFR and CD3 as a combined prognostic biomarker profile for OSCC patients in larger patient cohorts to strengthen our findings. If successful. this work has profound implications for potential treatment options for EGFR+ patients which generally have poor clinical outcomes.

## Data Availability Statement

The raw data supporting the conclusions of this article will be made available by the authors, upon reasonable request.

## Ethics Statement

The studies involving human participants were reviewed and approved by Institutional Review Board of the University of Iowa (IRB #201906841). The patients/participants provided their written informed consent to participate in this study.

## Author Contributions

Conception and design: AS, Development of methodology: WW, AS, KG-C, AR, MB, EL, Acquisition of data: WW, KG-C, AC, Analysis and interpretation of data: WW, AS, KG-C, AR, Writing, review, and/or revision of the manuscript: WW, AS, KG-C, AR, MB, EL, Study supervision: AS, AR. All authors contributed to the article and approved the submitted version.

## Funding

This work was funded by Merit Review Award #I01 BX004829-01 from the United States (U.S.) Department of Veterans Affairs Biomedical Laboratory Research and Development Service.

## Conflict of Interest

The authors declare that the research was conducted in the absence of any commercial or financial relationships that could be construed as a potential conflict of interest.

## Publisher’s Note

All claims expressed in this article are solely those of the authors and do not necessarily represent those of their affiliated organizations, or those of the publisher, the editors and the reviewers. Any product that may be evaluated in this article, or claim that may be made by its manufacturer, is not guaranteed or endorsed by the publisher.

## References

[1] Wong T Wiesenfeld D. Oral Cancer. Aust Dent J (2018) 63 Suppl 1:S91–s9. doi: 10.1111/adj.12594 29574808

[2] Gupta B Johnson NW Kumar N. Global Epidemiology of Head and Neck Cancers: A Continuing Challenge. Oncology (2016) 91(1):13–23. doi: 10.1159/000446117 27245686

[3] Bray F Ferlay J Soerjomataram I Siegel RL Torre LA Jemal A. Global Cancer Statistics 2018: GLOBOCAN Estimates of Incidence and Mortality Worldwide for 36 Cancers in 185 Countries. CA Cancer J Clin (2018) 68(6):394–424. doi: 10.3322/caac.21492 30207593

[4] Rivera C Oliveira AK Costa RAP De Rossi T Paes Leme AF. Prognostic Biomarkers in Oral Squamous Cell Carcinoma: A Systematic Review. Oral Oncol (2017) 72:38–47. doi: 10.1016/j.oraloncology.2017.07.003 28797460

[5] Argiris A Harrington KJ Tahara M Schulten J Chomette P Ferreira Castro A. Evidence-Based Treatment Options in Recurrent and/or Metastatic Squamous Cell Carcinoma of the Head and Neck. Front Oncol (2017) 7:72. doi: 10.3389/fonc.2017.00072 28536670 PMC5422557

[6] Kreppel M Nazarli P Grandoch A Safi AF Zirk M Nickenig HJ. Clinical and Histopathological Staging in Oral Squamous Cell Carcinoma - Comparison of the Prognostic Significance. Oral Oncol (2016) 60:68–73. doi: 10.1016/j.oraloncology.2016.07.004 27531875

[7] Costa V Kowalski LP Coutinho-Camillo CM Begnami MD Calsavara VF Neves JI. EGFR Amplification and Expression in Oral Squamous Cell Carcinoma in Young Adults. Int J Oral Maxillofac Surg (2018) 47(7):817–23. doi: 10.1016/j.ijom.2018.01.002 29395668

[8] Taghavi N Yazdi I. Prognostic Factors of Survival Rate in Oral Squamous Cell Carcinoma: Clinical, Histologic, Genetic and Molecular Concepts. Arch Iran Med (2015) 18(5):314–9. 25959914

[9] Bertics PJ Gill GN. Self-Phosphorylation Enhances the Protein-Tyrosine Kinase Activity of the Epidermal Growth Factor Receptor. J Biol Chem (1985) 260(27):14642–7. doi: 10.1016/S0021-9258(17)38618-0 2997217

[10] Bertics PJ Weber W Cochet C Gill GN. Regulation of the Epidermal Growth Factor Receptor by Phosphorylation. J Cell Biochem (1985) 29(3):195–208. doi: 10.1002/jcb.240290304 3001110

[11] Wee P Wang Z. Epidermal Growth Factor Receptor Cell Proliferation Signaling Pathways. Cancers (Basel) (2017) 9(5):52. doi: 10.3390/cancers9050052 PMC5447962 28513565

[12] Romano R Bucci C. Role of EGFR in the Nervous System. Cells (2020) 9(8):1887. doi: 10.3390/cells9081887 PMC7464966 32806510

[13] Sigismund S Avanzato D Lanzetti L. Emerging Functions of the EGFR in Cancer. Mol Oncol (2018) 12(1):3–20. doi: 10.1002/1878-0261.12155 29124875 PMC5748484

[14] Joseph SR Endo-Munoz L Gaffney DC Saunders NA Simpson F. Dysregulation of Epidermal Growth Factor Receptor in Actinic Keratosis and Squamous Cell Carcinoma. Curr Probl Dermatol (2015) 46:20–7. doi: 10.1159/000367959 25561202

[15] Kuttan NA Bhakthan NM. Epidermal Growth Factor Receptor (EGFR) in Oral Squamous Cell Carcinomas: Overexpression, Localization and Therapeutic Implications. Indian J Dent Res (1997) 8(1):9–18. 9495132

[16] Papagerakis S Pannone G Zheng L About I Taqi N Nguyen NP. Oral Epithelial Stem Cells - Implications in Normal Development and Cancer Metastasis. Exp Cell Res (2014) 325(2):111–29. doi: 10.1016/j.yexcr.2014.04.021 PMC4157336 24803391

[17] Miyaguchi M Olofsson J Hellquist HB. Immunohistochemical Study of Epidermal Growth Factor Receptor in Severe Dysplasia and Carcinoma *in Situ* of the Vocal Cords. Acta Otolaryngol (1991) 111(1):149–52. doi: 10.3109/00016489109137366 2014751

[18] Shin DM Ro JY Hong WK Hittelman WN. Dysregulation of Epidermal Growth Factor Receptor Expression in Premalignant Lesions During Head and Neck Tumorigenesis. Cancer Res (1994) 54(12):3153–9. 8205534

[19] Grobe A Eichhorn W Fraederich M Kluwe L Vashist Y Wikner J. Immunohistochemical and FISH Analysis of EGFR and its Prognostic Value in Patients With Oral Squamous Cell Carcinoma. J Oral Pathol Med (2014) 43(3):205–10. doi: 10.1111/jop.12111 24020871

[20] Laimer K Spizzo G Gastl G Obrist P Brunhuber T Fong D. High EGFR Expression Predicts Poor Prognosis in Patients With Squamous Cell Carcinoma of the Oral Cavity and Oropharynx: A TMA-Based Immunohistochemical Analysis. Oral Oncol (2007) 43(2):193–8. doi: 10.1016/j.oraloncology.2006.02.009 16854613

[21] Wheeler S Siwak DR Chai R LaValle C Seethala RR Wang L. Tumor Epidermal Growth Factor Receptor and EGFR PY1068 are Independent Prognostic Indicators for Head and Neck Squamous Cell Carcinoma. Clin Cancer Res (2012) 18(8):2278–89. doi: 10.1158/1078-0432.CCR-11-1593 PMC3430124 22351687

[22] Miyaguchi M Olofsson J Hellquist HB. Expression of Epidermal Growth Factor Receptor in Glottic Carcinoma and its Relation to Recurrence After Radiotherapy. Clin Otolaryngol Allied Sci (1991) 16(5):466–9. doi: 10.1111/j.1365-2273.1991.tb01041.x 1742894

[23] Khaznadar SS Khan M Schmid E Gebhart S Becker ET Krahn T. EGFR Overexpression is Not Common in Patients With Head and Neck Cancer. Cell Lines are Not Representative for the Clinical Situation in This Indication. Oncotarget (2018) 9(48):28965–75. doi: 10.18632/oncotarget.25656 PMC6034751 29989001

[24] Alterio D Marvaso G Maffini F Gandini S Chiocca S Ferrari A. Role of EGFR as Prognostic Factor in Head and Neck Cancer Patients Treated With Surgery and Postoperative Radiotherapy: Proposal of a New Approach Behind the EGFR Overexpression. Med Oncol (2017) 34(6):107. doi: 10.1007/s12032-017-0965-7 28452036

[25] Pedicini P Caivano R Jereczek-Fossa BA Strigari L Vischioni B Alterio D. Modelling the Correlation Between EGFr Expression and Tumour Cell Radiosensitivity, and Combined Treatments of Radiation and Monoclonal Antibody EGFr Inhibitors. Theor Biol Med Model (2012) 9:23. doi: 10.1186/1742-4682-9-23 22713695 PMC3502488

[26] Pedicini P Nappi A Strigari L Jereczek-Fossa BA Alterio D Cremonesi M. Correlation Between EGFr Expression and Accelerated Proliferation During Radiotherapy of Head and Neck Squamous Cell Carcinoma. Radiat Oncol (2012) 7:143. doi: 10.1186/1748-717X-7-143 22920680 PMC3537603

[27] Bossi P Resteghini C Paielli N Licitra L Pilotti S Perrone F. Prognostic and Predictive Value of EGFR in Head and Neck Squamous Cell Carcinoma. Oncotarget (2016) 7(45):74362–79. doi: 10.18632/oncotarget.11413 PMC5342059 27556186

[28] Monteiro LS Diniz-Freitas M Garcia-Caballero T Warnakulasuriya S Forteza J Fraga M. Combined Cytoplasmic and Membranous EGFR and P53 Overexpression Is a Poor Prognostic Marker in Early Stage Oral Squamous Cell Carcinoma. J Oral Pathol Med (2012) 41(7):559–67. doi: 10.1111/j.1600-0714.2012.01142.x 22417132

[29] Silva SD Alaoui-Jamali MA Hier M Soares FA Graner E Kowalski LP. Cooverexpression of ERBB1 and ERBB4 Receptors Predicts Poor Clinical Outcome in Pn+ Oral Squamous Cell Carcinoma With Extranodal Spread. Clin Exp Metastasis (2014) 31(3):307–16. doi: 10.1007/s10585-013-9629-y 24338375

[30] Ulanovski D Stern Y Roizman P Shpitzer T Popovtzer A Feinmesser R. Expression of EGFR and Cerb-B2 as Prognostic Factors in Cancer of the Tongue. Oral Oncol (2004) 40(5):532–7. doi: 10.1016/j.oraloncology.2003.11.004 15006627

[31] Shiraki M Odajima T Ikeda T Sasaki A Satoh M Yamaguchi A. Combined Expression of P53, Cyclin D1 and Epidermal Growth Factor Receptor Improves Estimation of Prognosis in Curatively Resected Oral Cancer. Modern Pathol (2005) 18(11):1482–9. doi: 10.1038/modpathol.3800455 16007067

[32] Monteiro L Ricardo S Delgado M Garcez F do Amaral B Lopes C. Phosphorylated EGFR at Tyrosine 1173 Correlates With Poor Prognosis in Oral Squamous Cell Carcinomas. Oral Dis (2014) 20(2):178–85. doi: 10.1111/odi.12087 23464360

[33] Monteiro LS Diniz-Freitas M Garcia-Caballero T Forteza J Fraga M. EGFR and Ki-67 Expression in Oral Squamous Cell Carcinoma Using Tissue Microarray Technology. J Oral Pathol Med (2010) 39(7):571–8. doi: 10.1111/j.1600-0714.2009.00876.x 20202087

[34] Na II Kang HJ Cho SY Koh JS Lee JK Lee BC. EGFR Mutations and Human Papillomavirus in Squamous Cell Carcinoma of Tongue and Tonsil. Eur J Cancer (2007) 43(3):520–6. doi: 10.1016/j.ejca.2006.09.025 17224267

[35] Nakata Y Uzawa N Takahashi K Sumino J Michikawa C Sato H. EGFR Gene Copy Number Alteration is a Better Prognostic Indicator Than Protein Overexpression in Oral Tongue Squamous Cell Carcinomas. Eur J Cancer (2011) 47(15):2364–72. doi: 10.1016/j.ejca.2011.07.006 21852109

[36] Ryott M Wangsa D Heselmeyer-Haddad K Lindholm J Elmberger G Auer G. EGFR Protein Overexpression and Gene Copy Number Increases in Oral Tongue Squamous Cell Carcinoma. Eur J Cancer (2009) 45(9):1700–8. doi: 10.1016/j.ejca.2009.02.027 PMC7294540 19332367

[37] Zhou C Wu Y Jiang L Li Z Diao P Wang D. Density and Location of CD3(+) and CD8(+) Tumor-Infiltrating Lymphocytes Correlate With Prognosis of Oral Squamous Cell Carcinoma. J Oral Pathol Med (2018) 47(4):359–67. doi: 10.1111/jop.12698 29469989

[38] Mukherjee G Bag S Chakraborty P Dey D Roy S Jain P. Density of CD3+ and CD8+ Cells in Gingivo-Buccal Oral Squamous Cell Carcinoma is Associated With Lymph Node Metastases and Survival. PloS One (2020) 15(11):e0242058. doi: 10.1371/journal.pone.0242058 33211709 PMC7676650

[39] Spector ME Bellile E Amlani L Zarins K Smith J Brenner JC. Prognostic Value of Tumor-Infiltrating Lymphocytes in Head and Neck Squamous Cell Carcinoma. JAMA Otolaryngology–Head Neck Surgery (2019) 145(11):1012–9. doi: 10.1001/jamaoto.2019.2427 PMC6735419 31486841

[40] Watanabe Y Katou F Ohtani H Nakayama T Yoshie O Hashimoto K. Tumor-Infiltrating Lymphocytes, Particularly the Balance Between CD8(+) T Cells and CCR4(+) Regulatory T Cells, Affect the Survival of Patients With Oral Squamous Cell Carcinoma. Oral Surg Oral Med Oral Pathol Oral Radiol Endod (2010) 109(5):744–52. doi: 10.1016/j.tripleo.2009.12.015 20303300

[41] Ni YH Zhang XX Lu ZY Huang XF Wang ZY Yang Y. Tumor-Infiltrating CD1a(+) DCs and CD8(+)/FoxP3(+) Ratios Served as Predictors for Clinical Outcomes in Tongue Squamous Cell Carcinoma Patients. Pathol Oncol Res (2020) 26(3):1687–95. doi: 10.1007/s12253-019-00701-5 31606786

[42] Diao P Jiang Y Li Y Wu X Li J Zhou C. Immune Landscape and Subtypes in Primary Resectable Oral Squamous Cell Carcinoma: Prognostic Significance and Predictive of Therapeutic Response. J Immunother Cancer (2021) 9(6):e002434. doi: 10.1136/jitc-2021-002434 34130988 PMC8208002

[43] Quan H Shan Z Liu Z Liu S Yang L Fang X. The Repertoire of Tumor-Infiltrating Lymphocytes Within the Microenvironment of Oral Squamous Cell Carcinoma Reveals Immune Dysfunction. Cancer Immunol Immunother (2020) 69(3):465–76. doi: 10.1007/s00262-020-02479-x PMC11027813 31950224

[44] Troiano G Rubini C Togni L Caponio VCA Zhurakivska K Santarelli A. The Immune Phenotype of Tongue Squamous Cell Carcinoma Predicts Early Relapse and Poor Prognosis. Cancer Med (2020) 9(22):8333–44. doi: 10.1002/cam4.3440 PMC7666743 33047888

[45] Lewis CE Pollard JW. Distinct Role of Macrophages in Different Tumor Microenvironments. Cancer Res (2006) 66(2):605–12. doi: 10.1158/0008-5472.CAN-05-4005 16423985

[46] Fukaya T Takagi H Taya H Sato K. DCs in Immune Tolerance in Steady-State Conditions. Methods Mol Biol (2011) 677:113–26. doi: 10.1007/978-1-60761-869-0_8 20941606

[47] Facciabene A Motz GT Coukos G. T-Regulatory Cells: Key Players in Tumor Immune Escape and Angiogenesis. Cancer Res (2012) 72(9):2162–71. doi: 10.1158/0008-5472.CAN-11-3687 PMC3342842 22549946

[48] Malek E de Lima M Letterio JJ Kim BG Finke JH Driscoll JJ. Myeloid-Derived Suppressor Cells: The Green Light for Myeloma Immune Escape. Blood Rev (2016) 30(5):341–8. doi: 10.1016/j.blre.2016.04.002 PMC6411302 27132116

[49] Gonzalez H Hagerling C Werb Z. Roles of the Immune System in Cancer: From Tumor Initiation to Metastatic Progression. Genes Dev (2018) 32(19-20):1267–84. doi: 10.1101/gad.314617.118 PMC6169832 30275043

[50] Lequerica-Fernandez P Suarez-Canto J Rodriguez-Santamarta T Rodrigo JP Suarez-Sanchez FJ Blanco-Lorenzo V. Prognostic Relevance of CD4(+), CD8(+) and FOXP3(+) TILs in Oral Squamous Cell Carcinoma and Correlations With PD-L1 and Cancer Stem Cell Markers. Biomedicines (2021) 9(6):653. doi: 10.3390/biomedicines9060653 34201050 PMC8227658

[51] Shimizu S Hiratsuka H Koike K Tsuchihashi K Sonoda T Ogi K. Tumor-Infiltrating CD8(+) T-Cell Density is an Independent Prognostic Marker for Oral Squamous Cell Carcinoma. Cancer Med (2019) 8(1):80–93. doi: 10.1002/cam4.1889 30600646 PMC6346233

[52] Wu J Zhang T Xiong H Zeng L Wang Z Peng Y. Tumor-Infiltrating CD4(+) Central Memory T Cells Correlated With Favorable Prognosis in Oral Squamous Cell Carcinoma. J Inflammation Res (2022) 15:141–52. doi: 10.2147/JIR.S343432 PMC8754505 35035226

[53] Balermpas P Michel Y Wagenblast J Seitz O Weiss C Rodel F. Tumour-Infiltrating Lymphocytes Predict Response to Definitive Chemoradiotherapy in Head and Neck Cancer. Br J Cancer (2014) 110(2):501–9. doi: 10.1038/bjc.2013.640 PMC3899751 24129245

[54] Nguyen N Bellile E Thomas D McHugh J Rozek L Virani S. Tumor Infiltrating Lymphocytes and Survival in Patients With Head and Neck Squamous Cell Carcinoma. Head Neck (2016) 38(7):1074–84. doi: 10.1002/hed.24406 PMC4900934 26879675

[55] Nordfors C Grun N Tertipis N Ahrlund-Richter A Haeggblom L Sivars L. CD8+ and CD4+ Tumour Infiltrating Lymphocytes in Relation to Human Papillomavirus Status and Clinical Outcome in Tonsillar and Base of Tongue Squamous Cell Carcinoma. Eur J Cancer (2013) 49(11):2522–30. doi: 10.1016/j.ejca.2013.03.019 23571147

[56] Gamboa-Dominguez A Dominguez-Fonseca C Quintanilla-Martinez L Reyes-Gutierrez E Green D Angeles-Angeles A. Epidermal Growth Factor Receptor Expression Correlates With Poor Survival in Gastric Adenocarcinoma From Mexican Patients: A Multivariate Analysis Using a Standardized Immunohistochemical Detection System. Modern Pathol (2004) 17(5):579–87. doi: 10.1038/modpathol.3800085 15073595

[57] Bourova-Flin E Derakhshan S Goudarzi A Wang T Vitte A-L Chuffart F. The Combined Detection of Amphiregulin, Cyclin A1 and DDX20/Gemin3 Expression Predicts Aggressive Forms of Oral Squamous Cell Carcinoma. Br J Cancer (2021) 125(8):1122–34. doi: 10.1038/s41416-021-01491-x PMC8505643 34290392

[58] Rajan A Gibson-Corley KN Choi AB Ofori-Amanfo GK Ten Eyck P Espinosa-Cotton M. Impact of Nuclear Interleukin-1 Alpha and EGFR Expression on Recurrence and Survival Outcomes in Oral Squamous Cell Carcinomas. J Oncol (2019) 2019:5859680. doi: 10.1155/2019/5859680 31320902 PMC6607721

[59] Polanska H Raudenska M Hudcova K Gumulec J Svobodova M Heger Z. Evaluation of EGFR as a Prognostic and Diagnostic Marker for Head and Neck Squamous Cell Carcinoma Patients. Oncol Lett (2016) 12(3):2127–32. doi: 10.3892/ol.2016.4896 PMC4998659 27602151

[60] Ma P He Q Li W Li X Han H Jin M. Anti-CD3 X EGFR Bispecific Antibody Redirects Cytokine-Induced Killer Cells to Glioblastoma *In Vitro* and In Vivo. Oncol Rep (2015) 34(5):2567–75. doi: 10.3892/or.2015.4233 26323605

[61] Chai AWY Lim KP Cheong SC. Translational Genomics and Recent Advances in Oral Squamous Cell Carcinoma. Semin Cancer Biol (2020) 61:71–83. doi: 10.1016/j.semcancer.2019.09.011 31542510

[62] Sales de Sá R Miranda Galvis M Mariz BALA Leite AA Schultz L Almeida OP. Increased Tumor Immune Microenvironment CD3+ and CD20+ Lymphocytes Predict a Better Prognosis in Oral Tongue Squamous Cell Carcinoma. Front Cell Dev Biol (2021) 8:622161. doi: 10.3389/fcell.2020.622161 33718347 PMC7951138

[63] Badoual C Hans S Rodriguez J Peyrard S Klein C Agueznay Nel H. Prognostic Value of Tumor-Infiltrating CD4+ T-Cell Subpopulations in Head and Neck Cancers. Clin Cancer Res (2006) 12(2):465–72. doi: 10.1158/1078-0432.CCR-05-1886 16428488

[64] Wu RQ Zhang DF Tu E Chen QM Chen W. The Mucosal Immune System in the Oral Cavity-an Orchestra of T Cell Diversity. Int J Oral Sci (2014) 6(3):125–32. doi: 10.1038/ijos.2014.48 PMC4170154 25105816

[65] Chatzopoulos K Sotiriou S Collins AR Kartsidis P Schmitt AC Chen X. Transcriptomic and Immunophenotypic Characterization of Tumor Immune Microenvironment in Squamous Cell Carcinoma of the Oral Tongue. Head Neck Pathol (2021) 15(2):509–22. doi: 10.1007/s12105-020-01229-w PMC8134601 33010009

[66] Heikkinen I Bello IO Wahab A Hagstrom J Haglund C Coletta RD. Assessment of Tumor-Infiltrating Lymphocytes Predicts the Behavior of Early-Stage Oral Tongue Cancer. Am J Surg Pathol (2019) 43(10):1392–6. doi: 10.1097/PAS.0000000000001323 31290758

[67] Lee HJ Kim JY Park IA Song IH Yu JH Ahn JH. Prognostic Significance of Tumor-Infiltrating Lymphocytes and the Tertiary Lymphoid Structures in HER2-Positive Breast Cancer Treated With Adjuvant Trastuzumab. Am J Clin Pathol (2015) 144(2):278–88. doi: 10.1309/AJCPIXUYDVZ0RZ3G 26185313

[68] Meehan K Leslie C Lucas M Jacques A Mirzai B Lim J. Characterization of the Immune Profile of Oral Tongue Squamous Cell Carcinomas With Advancing Disease. Cancer Med (2020) 9(13):4791–807. doi: 10.1002/cam4.3106 PMC7333861 32383556

[69] Schuler PJ Boeckers P Engers R Boelke E Bas M Greve J. EGFR-Specific T Cell Frequencies Correlate With EGFR Expression in Head and Neck Squamous Cell Carcinoma. J Transl Med (2011) 9:168. doi: 10.1186/1479-5876-9-168 21970318 PMC3198929

[70] Lu Y Jingyan G Baorong S Peng J Xu Y Cai S. Expression of EGFR, Her2 Predict Lymph Node Metastasis (LNM)-Associated Metastasis in Colorectal Cancer. Cancer biomark (2012) 11(5):219–26. doi: 10.3233/CBM-2012-00282 PMC13016206 23220854

[71] Christianto S Li KY Huang TH Su YX. The Prognostic Value of Human Papilloma Virus Infection in Oral Cavity Squamous Cell Carcinoma: A Meta-Analysis. Laryngoscope (2021). doi: 10.1002/lary.29996 34953144

[72] de Vicente JC Rodríguez-Santamarta T Rodrigo JP Blanco-Lorenzo V Allonca E García-Pedrero JM. PD-L1 Expression in Tumor Cells Is an Independent Unfavorable Prognostic Factor in Oral Squamous Cell Carcinoma. Cancer Epidemiol Biomarkers Prev (2019) 28(3):546–54. doi: 10.1158/1055-9965.EPI-18-0779 30487133

[73] Miranda-Galvis M Rumayor Piña A Sales de Sá R Almeida Leite A Agustin Vargas P Calsavara VF. PD-L1 Expression Patterns in Oral Cancer as an Integrated Approach for Further Prognostic Classification. Oral Dis (2021) 27(7):1699–710. doi: 10.1111/odi.13714 33169454

