# Supplementary figures and images for: Prognostic Role of Combined EGFR and Tumor-Infiltrating Lymphocytes in Oral Squamous Cell Carcinoma
Source: Front Oncol. 2022 Jul 25;12:885236. doi: 10.3389/fonc.2022.885236 (PMC9357911; doi:10.3389/fonc.2022.885236)

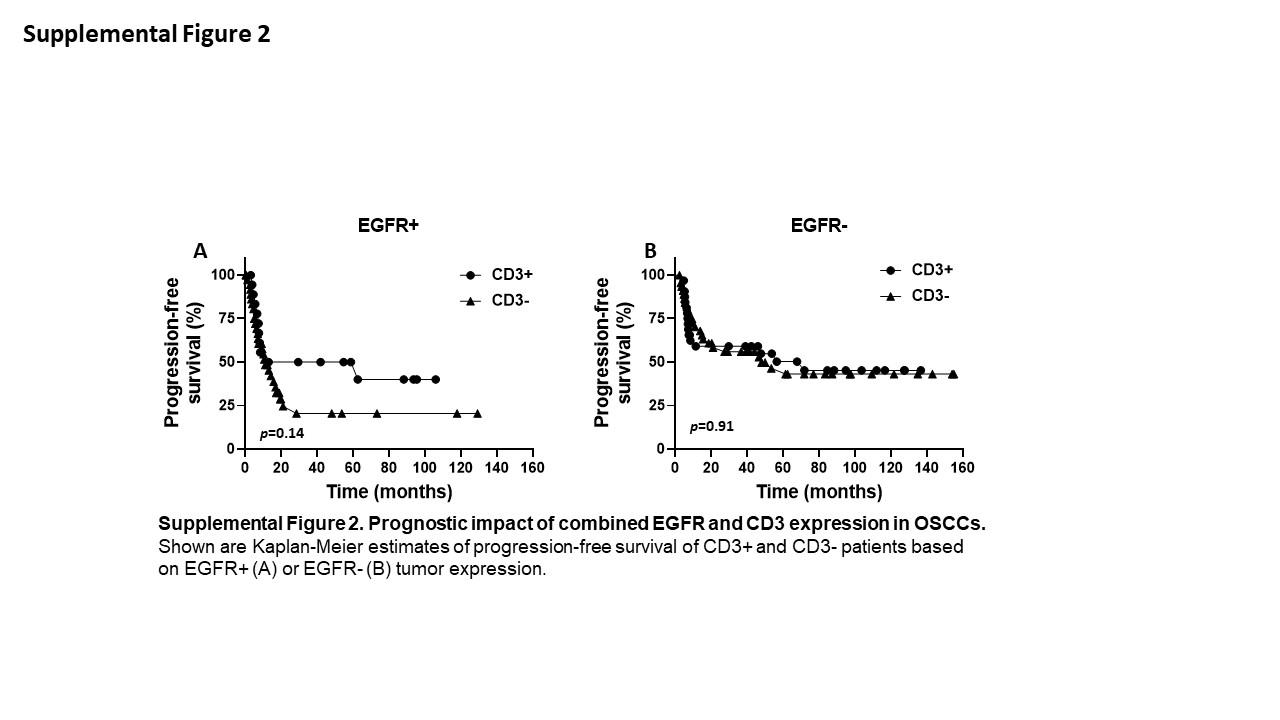

Supplement: Supplementary file 1 [file Image_1.jpeg]

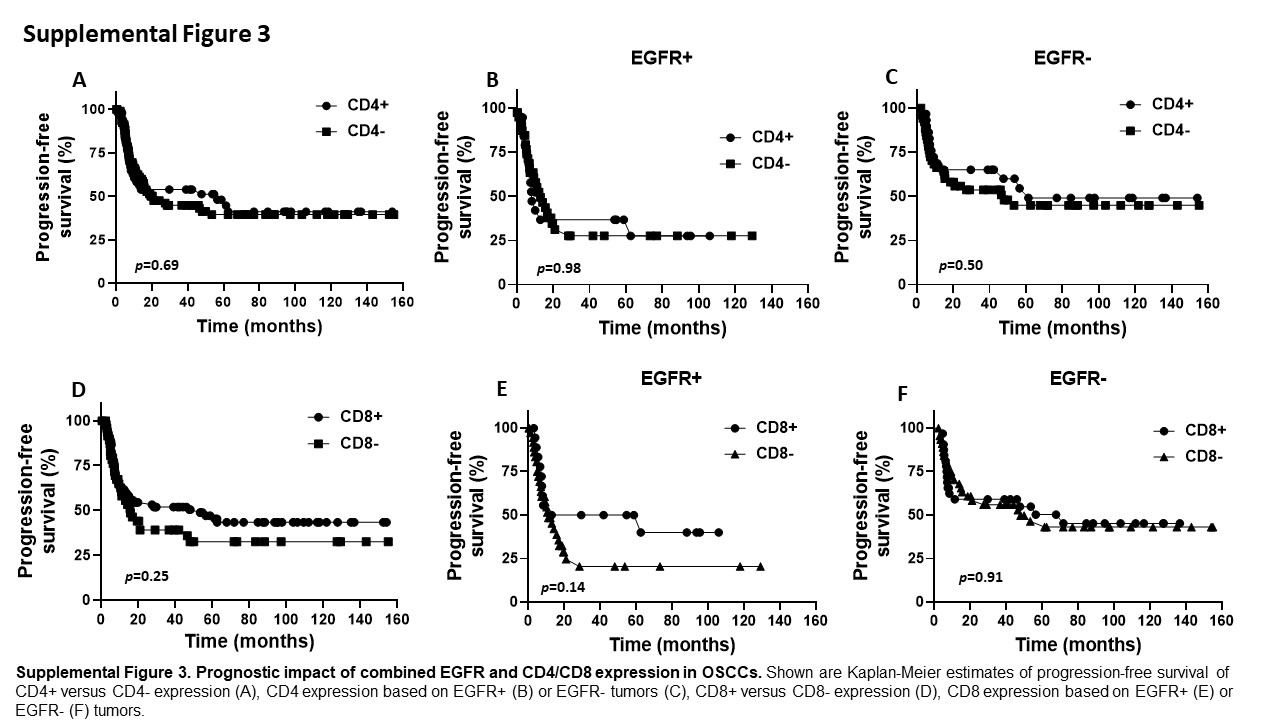

Supplement: Supplementary file 2 [file Image_2.jpeg]

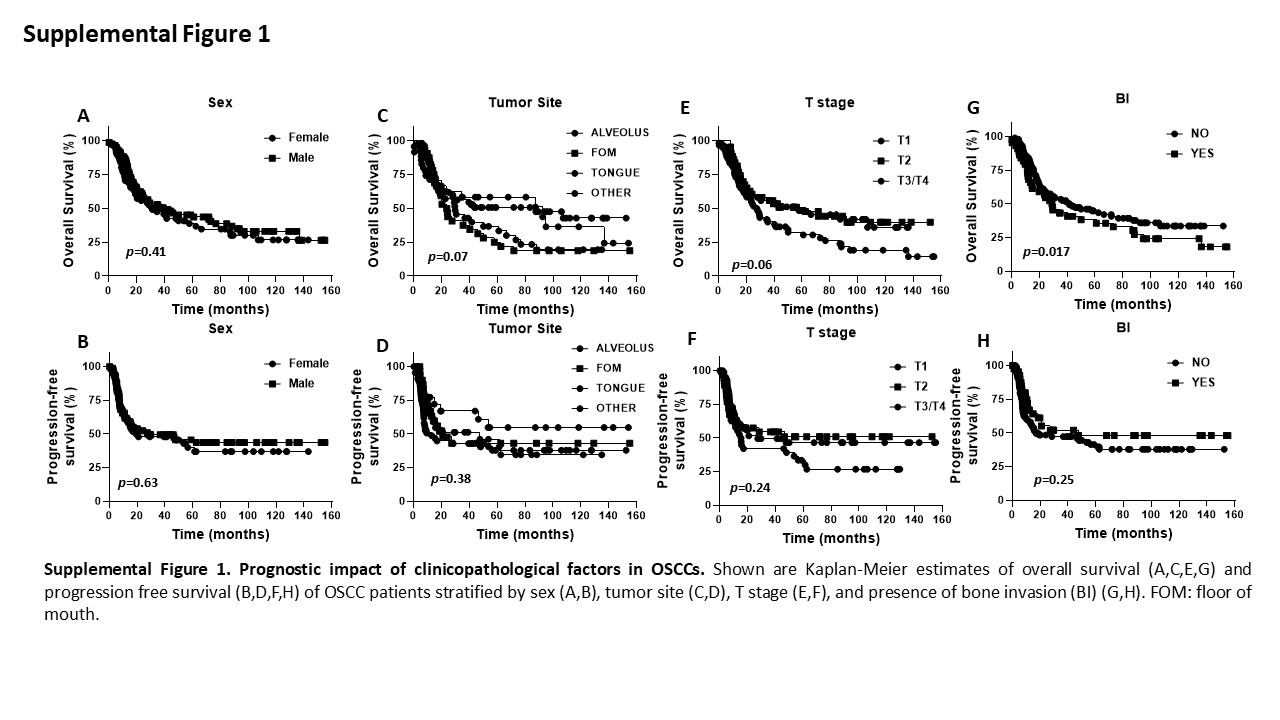

Supplement: Supplementary file 3 [file Image_3.jpeg]
